# Translation of predictive modeling and AI into clinics: a question of trust

**DOI:** 10.1007/s00330-021-07977-9

**Published:** 2021-04-25

**Authors:** Julian Caspers

**Affiliations:** University Düsseldorf, Medical Faculty, Department of Diagnostic and Interventional Radiology, D-40225 Düsseldorf, Germany

During the last decade, data science technologies such as artificial intelligence (AI) and radiomics have emerged strongly in radiologic research. Radiomics refers to the (automated) extraction of a large number of quantitative features from medical images [1]. A typical radiomics workflow involves image acquisition and segmentation as well as feature extraction and prioritization/reduction as preparation for its ultimate goal, which is predictive modeling [2]. This final step is where radiomics and AI typically intertwine to build a gainful symbiosis.

In recent years, the field of medical imaging has seen a rising number of publications on radiomics and AI applications with increasingly refined methodologies [3, 4]. Formulation of best practice white papers and quality criteria for publications on predictive modeling like the TRIPODS [5] or CLAIM [6] criteria have substantially promoted this qualitative gain. Consequently, relevant methodological approaches advancing generalizability of predictive models are increasingly being observed in recent publications, e.g., the accurate composition of representative and unbiased datasets, avoidance of data leakage, the incorporation of (nested) cross-validation approaches for model development, particularly on small datasets, or the use of independent, external test samples. In this regard, the work of Song et al [7] on a clinical-radiomics nomogram for prediction of functional outcome in intracranial hemorrhage published in the current issue of *European Radiology* is just one example for the general trend.

However, in contrast to the rising utilization and importance of predictive modeling in medical imaging research, these technologies have not been widely adopted in clinical routine. Beside regulatory, medicolegal, or ethical issues, one of the major hurdles for a broad usage of AI and predictive models is the lack of trust in these technologies by medical practitioners, healthcare stakeholders, and patients. After more than a decade of scientific progress on AI and predictive modeling in medical imaging, we should now take the opportunity to focus our research on the trustworthiness of AI and predictive modeling in order to trailblaze their translation into clinical practice.

Several prospects could enhance trustworthiness of predictive models for clinical use. One of the main factors will be transparency on their reliability in real-world applications. Large multicentric prospective trials will be paramount to assess and validate the performance and especially generalizability of predictive models in a robust and minimally biased fashion. Additionally, benchmarking of AI tools by independent institutions on external heterogeneous real-world data would provide transparency on model performances and enhance trust.

In general, trust in new technologies is severely influenced by the comprehensibility of these techniques for their users. In the field of predictive modeling, this topic is often described with the term “explainable AI,” which is being increasingly considered in current research [8]. Explainable AI seeks to unravel the “black-box” nature of many predictive models, including artificial neural networks, by making decision processes comprehendible, e.g., by revealing the features that drive their decisions. Trust in predictive models will therefore substantially increase, when models are developed transparently and AI systems made comprehensible. Another issue of current AI tools is that they mainly incorporate narrow AI, i.e., they address only one very specific task. We are currently miles, if not light-years away, from building real strong AI, that is, artificial intelligence having the capacity to learn any intellectual task that a human being can. However, building more comprehensive AI systems solving multiple predictive tasks might enhance their trustworthiness for users. For example, a user might be inclined to follow thoughts along the line of “I have good experience in this system predicting the outcome of disease X, then it will likely perform well in prediction of outcome in disease Y and disease Z.” Another point that could increase trustworthiness of AI systems might be transparency on their level of confidence or uncertainty on a specific prediction. Currently, many predictive models discussed in recent literature yield hard binary classifications, i.e., they assign a dataset exclusively to one of two or more classes, for example, diseased vs. not diseased or good outcome vs. unfavorable outcome. If results from predictive models would also include an indication of certainty on classification, model-based decisions would potentially be perceived as more genuine or human-like, which could increase their trustworthiness and also their applicability in a clinical setting [9]. Such probabilistic classification approaches can for example be realized with methods like probability calibration or fuzzy classifiers. Additionally, the adjustment of pretrained models to local conditions should be more strongly considered in AI research. Individual fine-tuning of models, e.g., by applying techniques from domain adaptation and transfer learning [10], would allow for harmonization of different scanners, imaging protocols, or patient populations and avoid biases between the data used to train the model and at the site of usage. If predictive models would be tailored specifically to the local domain of application in such a way, this would clearly enhance their reliability and trustworthiness. Last but not least, the seamless integration of AI into radiologic workflows will be vital for its wide utilization. Close-knit cooperation between researchers, developers, and vendors promoting direct inclusion of predictive models into PACS and image-generating systems as well as upcoming AI marketplaces may strongly facilitate AI adoption.

In conclusion, time is ripe to focus research on the translation of predictive modeling into clinical practice and on approaches to enhance its trustworthiness in a clinical context. The prophecy of AI as a game-changer for radiology is already ubiquitous; it is now up to us to make it happen.

## Declarations

### Guarantor

The scientific guarantor of this publication is Julian Caspers.

### Statistics and Biometry

No complex statistical methods were necessary for this paper.

### Methodology

- editorial comment
